# Cytoplasmic–Nuclear Incompatibility Between Wild Isolates of *Caenorhabditis nouraguensis*

**DOI:** 10.1534/g3.116.037101

**Published:** 2017-01-05

**Authors:** Piero Lamelza, Michael Ailion

**Affiliations:** * Molecular and Cellular Biology Program, University of Washington, Seattle, Washington 98195; † Department of Biochemistry, University of Washington, Seattle, Washington 98195

**Keywords:** hybrid incompatibility, speciation, cytoplasmic–nuclear incompatibility, mitochondria, *Caenorhabditis*

## Abstract

How species arise is a fundamental question in biology. Species can be defined as populations of interbreeding individuals that are reproductively isolated from other such populations. Therefore, understanding how reproductive barriers evolve between populations is essential for understanding the process of speciation. Hybrid incompatibility (for example, hybrid sterility or lethality) is a common and strong reproductive barrier in nature. Here we report a lethal incompatibility between two wild isolates of the nematode *Caenorhabditis nouraguensis*. Hybrid inviability results from the incompatibility between a maternally inherited cytoplasmic factor from each strain and a recessive nuclear locus from the other. We have excluded the possibility that maternally inherited endosymbiotic bacteria cause the incompatibility by treating both strains with tetracycline and show that hybrid death is unaffected. Furthermore, cytoplasmic–nuclear incompatibility commonly occurs between other wild isolates, indicating that this is a significant reproductive barrier within *C. nouraguensis*. We hypothesize that the maternally inherited cytoplasmic factor is the mitochondrial genome and that mitochondrial dysfunction underlies hybrid death. This system has the potential to shed light on the dynamics of divergent mitochondrial–nuclear coevolution and its role in promoting speciation.

How species arise is a fundamental and still unanswered question in biology. Under the biological species concept, species consist of populations of interbreeding individuals that are reproductively isolated from other such populations (Mayr 1942). Thus, to understand speciation, we must learn how reproductive barriers evolve between populations. Postzygotic reproductive barriers are commonly found in nature, and occur when hybrid progeny are relatively unfit in comparison to their parents and serve as inefficient bridges for gene flow between populations. Hybrids can be extrinsically unfit, in that they are maladapted to their environment (for example, hybrids exhibit an intermediate phenotype which is unfit in parental environments) or intrinsically unfit, in that they are developmentally abnormal (for example, hybrids are sterile or inviable) (Coyne and Orr 2004).

The Bateson–Dobzhansky–Muller (BDM) model hypothesizes that hybrids are intrinsically unfit due to incompatible gene combinations. In its simplest form, the model predicts that at least two genetic loci, each having evolved independently in one of two divergent lineages, have deleterious epistatic interactions in hybrids. This model has gained support by the molecular identification of genes required for hybrid dysfunction in several genera (Presgraves 2010). Identifying these genes and the natural forces that drive their evolution is one of the major objectives of speciation genetics. Darwin suggested that differential ecological adaptation by natural selection was the major driving force for speciation. Some of the molecularly identified incompatibility genes do indeed show signs of selection (Ting *et al.* 1998; Presgraves *et al.* 2003; Barbash *et al.* 2004; Brideau *et al.* 2006; Oliver *et al.* 2009; Chae *et al.* 2014; Phadnis *et al.* 2015), but these genes do not always have a clear role in promoting ecological adaptation (Tao *et al.* 2001; Ferree and Barbash 2009; Phadnis and Orr 2009; Seidel *et al.* 2011). However, there are currently only a handful of known incompatibility genes from a limited number of genera. Additional studies from a wider range of taxa are needed to gain a better understanding of the evolutionary forces that drive speciation.

Some studies on the genetic basis of hybrid incompatibility have focused on strong postzygotic reproductive barriers between well-defined species, and show that many genetic variants contribute to dysfunction of hybrids (Coyne and Orr 1998). These studies are valuable, but it is difficult to determine the dynamics of the accumulation of such variants or their relative roles in initiating speciation. For example, theoretical work indicates that the number of genetic incompatibilities increases greater than linearly with the number of genetic differences between two lineages (Orr 1995). Therefore, a small number of genetic incompatibilities may initially reduce gene flow and promote genetic divergence between populations, whereas others evolve after strong reproductive barriers have already been established. Given this, studies of incomplete postzygotic barriers between young species or divergent populations within species are essential to understand the evolutionary forces that initiate speciation.

Despite the paucity of molecularly identified incompatibility genes, the segregation of deleterious phenotypes in a number of interspecific hybridizations indicates that incompatibilities between cytoplasmic and nuclear genomes occur frequently (Ellison and Burton 2008; Ellison *et al.* 2008; Sambatti *et al.* 2008; Arnqvist *et al.* 2010; Ross *et al.* 2011; Aalto *et al.* 2013). Furthermore, several studies have definitively mapped these incompatibility loci to the mitochondrial genome and nuclear genes with mitochondrial functions (Lee *et al.* 2008; Chou *et al.* 2010; Luo *et al.* 2013; Meiklejohn *et al.* 2013; Huang *et al.* 2015). Aerobic eukaryotic organisms rely on mitochondria to generate energy required for diverse biological processes. The mitochondrial genome encodes a small fraction of the mitochondrial proteins. Nuclear genes encode the majority of mitochondrial proteins and are also required for the proper replication, transcription, and translation of mtDNA (Gustafsson *et al.* 2016). Given the interdependence of the nuclear and mitochondrial genomes, they are expected to coevolve by the accumulation of compatible mutations that maintain mitochondrial function. By extension, distinct lineages that undergo unique mitochondrial–nuclear coevolution may be incompatible and result in mitochondrial dysfunction. Several theories have been proposed to explain what drives the rapid coevolution of these two genomes, including adaptation to different carbon sources (Lee *et al.* 2008), arms races between the genomes caused by genetic conflict over the relative fitness of males and females (Fujii *et al.* 2011), and the accumulation of deleterious mtDNA mutations and the evolution of compensatory nuclear variants that rescue mitochondrial function (Rand *et al.* 2004; Oliveira *et al.* 2008; Osada and Akashi 2012). However, given the scarcity of molecularly identified cases of mitochondrial–nuclear incompatibilities, additional studies are required to form more complete theories regarding the forces that drive their evolution.

Here we report incompatibility between the cytoplasmic and nuclear genomes of two distinct wild isolates of the male-female nematode *Caenorhabditis nouraguensis*. Cytoplasmic–nuclear incompatibility is not specific to these two strains, but is also observed upon hybridization of other distinct wild isolates of *C. nouraguensis*, indicating that this is a naturally widespread reproductive barrier within the species. This cytoplasmic–nuclear incompatibility may provide an excellent opportunity for a detailed study of mitochondrial–nuclear incompatibility, the forces that drive the coevolution of these genomes, and their possible role in speciation.

## Materials and Methods

### Strain isolation and maintenance

All strains of *C. nouraguensis* used in this study were derived from single gravid females isolated in 2009 or 2011 from rotten fruit or flowers found in French Guiana (Kiontke *et al.* 2011; Félix *et al.* 2013; C. Braendle, personal communication), and have not been subjected to further inbreeding. Strains were kindly provided by Marie-Anne Félix (“JU” prefix) and Christian Braendle (“NIC” prefix). Strain stocks were stored at −80°. Thawed strains were maintained at 25° on standard NGM plates spread with a thin lawn of OP50 bacteria (Brenner 1974).

### Hybridizing JU1825 and NIC59

To quantify inviability, we crossed one virgin L4 female and male, with 10–15 replicates for each cross. The edge of each plate was coated with a palmitic acid solution (10 mg/ml in 95% ethanol) and allowed to air dry, resulting in a physical barrier that helps prevent worms from leaving the plate’s surface. The plates were placed at 25° overnight, during which the worms matured to adulthood and began mating. The next day, each female–male couple was placed onto a new plate streaked with OP50 and rimmed with palmitic acid. Each couple was then allowed to mate and lay eggs for 5 hr at 25°, and then were permanently removed. The embryos laid within those 5 hr were counted immediately. Approximately 17 hr later, we counted the number of embryos that failed to hatch per plate. These unhatched embryos were scored as dead since *C. nouraguensis* embryogenesis is normally completed within 13 hr at 25° (data not shown). We defined the percentage of embryonic lethality as the number of unhatched embryos divided by the total number of embryos laid. Approximately 20 hr later, we placed the plates at 4° for 1 hr and then counted the number of healthy L4 larvae and young adults per plate. We defined the percentage of viable progeny as the total number of L4 larvae and young adults divided by the total number of embryos laid.

### Determining cytoplasmic–nuclear compatibility between various strains of C. nouraguensis

The genotype of a strain is designated by the following nomenclature: (cytoplasmic genotype); nuclear genotype. The cytoplasmic genotype indicates genetic elements that are inherited only maternally, such as the mitochondrial genome. To test for an incompatibility between one strain’s cytoplasm and another strain’s nuclear genome, we compared the viabilities of backcrosses that differ only in the F1 hybrid female’s cytoplasmic genotype (for example, (NIC59); NIC59/JU1837 F1 female × JU1837 male *vs.* (JU1837); NIC59/JU1837 F1 female × JU1837 male, Figure 3B). We performed a Fisher’s exact test to determine whether there were significant differences in the proportions of viable and inviable F2 progeny between the two types of crosses. We also calculated the relative viability of the two crosses (for example, the percent viability of the (NIC59); NIC59/JU1837 F1 female × JU1837 male cross divided by the percent viability of (JU1837); NIC59/JU1837 F1 female × JU1837 male cross). Cytoplasmic–nuclear combinations that show a statistically significant difference in viabilities between the two types of crosses and a relative viability <1 were considered to be cytoplasmic–nuclear incompatibilities. Three biological replicates were performed for each cytoplasmic–nuclear combination except for JU1825 cytoplasmic–NIC24 nuclear and JU1825 cytoplasmic–NIC54 nuclear, which have four replicates each. For each biological replicate, 10 F1 hybrid L4 females were crossed to 10 L4 males on the same plate overnight at 25°. The next day, they were moved to a new plate and allowed to lay embryos at 25° for 1 hr. The parents were then removed and the percent viable progeny and embryonic lethality were calculated as described in the previous section of the *Materials and Methods*. The heat map used to visualize the median relative viability for each cytoplasmic–nuclear combination was made using the heatmap.2 function from the gplot package in R.

### Molecular methods

To determine if either JU1825 or NIC59 are infected with *Wolbachia*, we performed PCR on crude lysates of both strains using degenerate primers targeted against two genes that are conserved in *Wolbachia* (Baldo *et al.* 2006). Specifically, we attempted to detect *gatB* (gatB_F1 with M13 adapter, TGTAAAACGACGGCCAGTGAKTTAAAYCGYGCAGGBGTT, and gatB_R1 with M13 adapter, CAGGAAACAGCTATGACCTGGYAAYTCRGGYAAAGATGA) and *fbpA* (fbpA_F3, GTTAACCCTGATGCYYAYGAYCC, and fbpA_R3, TCTACTTCCTTYGAYTCDCCRCC). As controls, we performed PCR on squash preps of *Drosophila melanogaster* w^1118^ mutant strains (Bloomington stock number 3605) that were infected or not infected with *Wolbachia*. *Drosophila melanogaster* strains were kindly provided by the laboratories of H. Malik and L. Pallanck.

### Tetracycline treatment of JU1825 and NIC59

Both JU1825 and NIC59 were passaged on 50 μg/ml tetracycline NGM plates streaked with OP50 for nine generations. Both strains were treated by crossing 10 L4 females and 10 L4 males on a fresh tetracycline plate each generation. Tetracycline plates were made by allowing NGM plates with OP50 lawns to soak up a mixture of tetracycline and 1× M9. The plates were left uncovered at room temperature until dry, and then used the following day.

### Statistics

*P*-values were determined using R (v 3.2.5). Several statistical tests were used (Kruskal–Wallis test followed by Dunn’s test, and Fisher’s exact test). When we performed several comparisons on the same dataset, we used the Bonferroni method to correct *P*-values for multiple testing. Most plots were made using the ggplot2 package in R.

### Data availability

The authors state that all data necessary for confirming the conclusions presented in the article are represented fully within the article and Supplemental Material.

## Results

### Two strains of C. nouraguensis exhibit F2 hybrid breakdown

**Figure 1:**
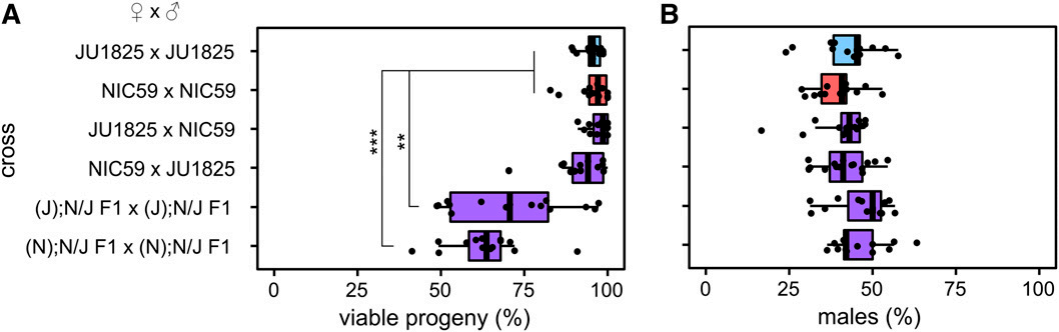
JU1825 and NIC59 exhibit F2 hybrid breakdown. Crosses are listed on the *y*-axis. Letters in parentheses to the left of a semicolon denote the cytoplasmic genotype of an individual (for example, “(J)” individuals have a JU1825 cytoplasmic genotype), while letters to the right of a semicolon denote the genotypes of all autosomal loci (that is, “N/J” individuals are heterozygous NIC59/JU1825 throughout the autosomes). (A) Only (J); N/J F1 × (J); N/J F1 and (N); N/J F1 × (N); N/J F1 crosses exhibit a significant decrease in the percentage of viable progeny (*P* < 0.01 and *P* < 0.001, respectively). (B) There are no significant differences in the percentages of viable males between crosses (*P* > 0.05). *N* = 14 or 15 plates per cross. All *P*-values were calculated by a Kruskal–Wallis test followed by Dunn’s test.

Two strains of *C. nouraguensis*, JU1825 and NIC59, were derived from single gravid females that were isolated ∼112 km apart in French Guiana (Kiontke *et al.* 2011). Both of these strains were designated as *C. nouraguensis* based on having highly similar ITS2 rDNA sequences (a good species barcode within the *Caenorhabditis* genus), and because they produced many viable F1 offspring when crossed (Kiontke *et al.* 2011; Félix *et al.* 2014). We found that both strains produce high numbers of viable progeny in intrastrain crosses. We also confirmed the previous finding of F1 hybrid viability by crossing NIC59 females to JU1825 males, and vice versa, showing that the F1 hybrids resulting from these interstrain crosses exhibit levels of viability comparable to those seen in intrastrain crosses (Figure 1A).

However, not all reproductive barriers act in the F1 generation. There are many cases of F2 hybrid breakdown, in which reduction of hybrid fitness is seen in the F2 generation due to recessive incompatibility loci (Masly *et al.* 2006; Bikard *et al.* 2009; Dey *et al.* 2012, 2014; Stelkens *et al.* 2015). To test for F2 hybrid inviability, we mated hybrid F1 siblings derived from either JU1825 female × NIC59 male crosses, or from NIC59 female × JU1825 male crosses, and assayed the F2 generation for reductions in fitness. These F1 hybrids are referred to as “(J); N/J” and “(N); N/J”, respectively, where the genotype is designated by the following nomenclature: (cytoplasmic genotype); nuclear genotype. The cytoplasmic genotype indicates genetic elements that are inherited only maternally, such as the mitochondrial genome. We found that both types of F1 sibling crosses resulted in a significant decrease in the percentage of viable progeny, with on average only 71 and 63% of F2 embryos maturing to the L4 or young adult stage (Figure 1A). These results indicate that there are divergent genomic loci between NIC59 and JU1825 that cause inviability only when they become homozygous in F2 hybrids. Additionally, there is no difference in sex-specific mortality in hybrids in comparison to intrastrain crosses (Figure 1B), implying that these loci are autosomally linked, as we show later.

### Incompatibilities between cytoplasmic and nuclear genomes cause F2 inviability

To further understand the genetic architecture of hybrid breakdown between JU1825 and NIC59, we tested whether maternally or paternally inherited factors are required for F2 inviability. We reasoned that backcrossing F1 females to parental males would test whether maternal factors are required for reduced hybrid fitness, while backcrossing F1 males to parental females would test whether paternal factors are required. For example, backcrossing F1 hybrid females to JU1825 males will result in an F2 population with a 50% chance of being heterozygous (NIC59/JU1825) and a 50% chance of being homozygous (JU1825/JU1825) for any given autosomal locus. Therefore, this cross will test for maternally deposited NIC59 factors that are incompatible with homozygous JU1825 autosomal loci. The same logic can be applied to crosses of F1 hybrid males to parental strain females.

**Figure 2:**
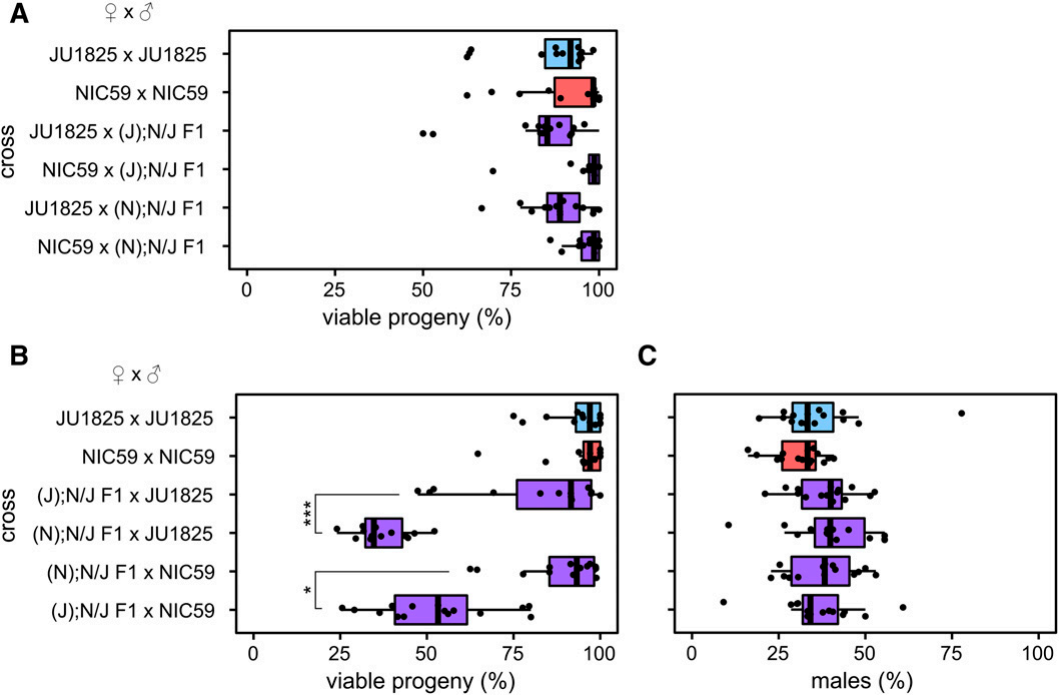
F2 inviability involves a maternal cytoplasmic effect. (A) There is no significant difference in the percentage of viable progeny between any of the F1 hybrid male backcrosses and intrastrain crosses (*P* > 0.05). (B) Backcrossing hybrid females to parental strain males reveals that only (N); N/J F1 female × JU1825 male crosses and (J); N/J F1 female × NIC59 male crosses exhibit a significant decrease in the percentage of viable progeny in comparison to intrastrain crosses (*P* < 0.001). (N); N/J F1 female × JU1825 male crosses have significantly decreased viability in comparison to (J); N/J F1 female × JU1825 male crosses (*P* < 0.001). Additionally, (J); N/J F1 female × NIC59 male crosses consistently have significantly decreased viability in comparison to (N); N/J F1 female × NIC59 male crosses (*P* < 0.05). The viability of (J); N/J F1 female × JU1825 males can differ significantly between experiments (one of three biological replicates is shown here, see Figure S1 for the other two). (C) There are no significant differences in the proportion of viable males between the crosses (*P* > 0.05). *N* = 14 or 15 plates per cross. All *P*-values were calculated by a Kruskal–Wallis test followed by Dunn’s test.

All backcrosses of F1 hybrid males to parental strain females resulted in levels of F2 viability similar to those observed in parental strains. Therefore, paternal factors do not have a major effect on F2 inviability (Figure 2A). Only two crosses consistently resulted in significantly reduced viability. The first is when (N); N/J F1 females were crossed to JU1825 males, with on average only 36% of F2 hybrids maturing to the L4 or young adult stage. This cross implies that there are maternally derived NIC59 factors distributed to F2 embryos, and these factors are incompatible with recessive JU1825 nuclear loci. The second is when (J); N/J F1 females are crossed to NIC59 males, with on average only 52% of the F2 hybrids maturing to the L4 or young adult stage (Figure 2B). This cross implies that there are also maternally derived JU1825 factors distributed to F2 embryos, and these factors are incompatible with recessive NIC59 nuclear loci. The viability of (J); N/J F1 female × JU1825 male crosses can also be significantly reduced in comparison to intrastrain crosses, but varies within and between experiments (Supplemental Material, Figure S1).

The F1 female backcross experiments show that almost identical crosses, which differ only in the cytoplasmic genotype of the F1 female, have significantly different rates of F2 viability. For instance, (N); N/J F1 female × JU1825 male crosses consistently have significantly lower F2 viability than (J); N/J F1 female × JU1825 male crosses (Figure 2 and Figure S1). Similarly, (J); N/J F1 female × NIC59 male crosses consistently have significantly lower F2 viability than (N); N/J F1 female × NIC59 male crosses (Figure 2B). The F1 hybrid females in these pairs of crosses are expected to be genotypically identical at all nuclear loci, suggesting that something other than the F1 nuclear genome encodes maternal factors that lead to F2 inviability.

One model to explain these backcrosses is that the mitochondrial genome is the maternally inherited factor that is incompatible with recessive nuclear loci in the F2 generation. For example, all F2 progeny from (N); N/J F1 female × JU1825 male crosses will inherit only NIC59 mtDNA, which may be incompatible with nuclear loci homozygous (or hemizygous) for JU1825 alleles, resulting in inviability (Figure 6A). In comparison, all F2 progeny from (J); N/J F1 female × JU1825 male crosses will inherit only JU1825 mtDNA, which should be compatible with the JU1825 nuclear genome and therefore not result in the same inviability. The same logic can be applied to the (J); N/J F1 female × NIC59 male and (N); N/J F1 female × NIC59 male crosses. We hypothesize that F2 inviability is the result of two mitochondrial–nuclear incompatibilities, one between the NIC59 mitochondrial genome and recessive JU1825 nuclear loci, and another between the JU1825 mitochondrial genome and recessive NIC59 nuclear loci.

### The nuclear incompatibility loci are linked to autosomes

Nematodes commonly have an XX (female) and XO (male) sex-determining mechanism (Pires-daSilva 2007). The F1 hybrid female backcross experiments reveal that there is no difference in sex-specific mortality in hybrids in comparison to intrastrain crosses (Figure 2C). However, given the expected genotypes of their F2 populations, these backcrosses on their own do not allow us to determine whether the nuclear incompatibility loci are autosomally or X-linked. In the previous section, we concluded that the inviability of the F2 progeny derived from (N); N/J F1 female × JU1825 male crosses is the result of a genetic incompatibility between the NIC59 cytoplasmic genome and nuclear loci homozygous (or hemizygous) for JU1825 alleles. If this is true, it is reasonable to assume that the same genetic incompatibility occurs in (N); N/J F1 female × (N); N/J F1 male crosses (Figure 1A). In this F1 sibling cross, if the JU1825 nuclear incompatibility locus were autosomally linked, both sexes would suffer equal rates of inviability. However, if the nuclear incompatibility locus were linked to the X-chromosome, then we would expect a significant decrease in the proportion of viable males in comparison to intrastrain crosses (Figure S2). However, we observe no significant difference in the proportion of viable males for the (N); N/J F1 female × (N); N/J F1 male cross (Figure 1B). Therefore, given the data from the F1 female backcrosses and the F1 sibling crosses, we conclude that the JU1825 nuclear incompatibility locus is autosomally linked. A similar line of reasoning indicates that the NIC59 nuclear incompatibility locus is also autosomally linked.

### Endosymbiotic bacteria do not cause hybrid inviability

We hypothesize that mitochondrial genomes are responsible for the cytoplasmic component of the hybrid incompatibility between NIC59 and JU1825. However, we also considered whether endosymbiotic bacteria of the *Rickettsiales* order could be involved. Within this order, bacteria of the *Wolbachia* genus are known to infect certain species of nematodes, and are transmitted to host progeny through female gametes (Werren *et al.* 2008). Furthermore, hybrid lethality in interstrain and interspecies crosses is sometimes caused by infection with divergent *Wolbachia* strains (Bourtzis *et al.* 1996; Bordenstein *et al.* 2001). However, we failed to detect conserved genes typically used to genotype diverse strains of *Wolbachia* in either JU1825 or NIC59 using PCR with degenerate primers (Figure S3A). Additionally, treatment of both strains with tetracycline for nine generations failed to rescue hybrid inviability (Figure S3B). Endosymbiotic bacteria within the *Rickettsiales* order are typically susceptible to tetracycline (McOrist 2000; Darby *et al.* 2015). Thus, endosymbiotic bacteria are unlikely to cause the reproductive barrier between NIC59 and JU1825.

### Cytoplasmic–nuclear incompatibility is common within C. nouraguensis

**Figure 3:**
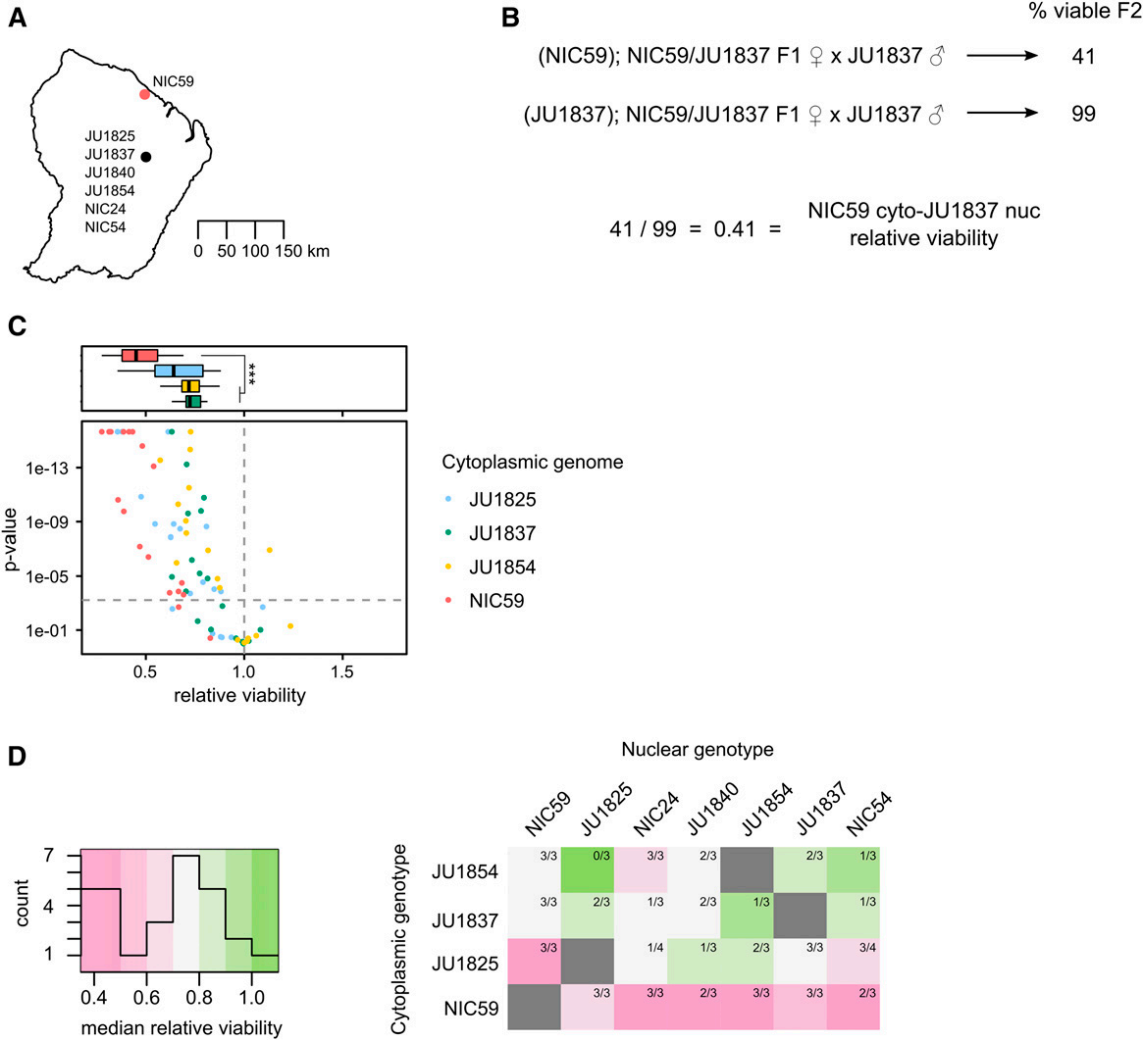
Cytoplasmic–nuclear incompatibility is widespread within *C. nouraguensis*. (A) A map depicting the two major sites where the strains used in this study were collected in French Guiana. GPS coordinates for NIC54 were obtained from C. Braendle (personal communication), while the other six were obtained from Kiontke *et al.* (2011) and Félix *et al.* (2013). Strains in the southern collection site were collected from distinct rotten fruits or flowers within 2 km of each other and are represented as a single point. (B) To determine whether a particular cytoplasmic–nuclear combination is incompatible, we tested for statistical differences in viability between the F1 female backcross that combines heterotypic cytoplasmic and nuclear genotypes (top cross) and the backcross that combines homotypic cytoplasmic and nuclear genotypes (bottom cross, see *Materials and Methods*). We also calculated the relative viability of the first cross to the second. (C) A scatter plot depicting all the cytoplasmic–nuclear compatibility tests performed. Each point corresponds to a single replicate of a certain cytoplasmic–nuclear combination. Points above the horizontal dashed gray line indicate statistically significant differences in viability between the two types of crosses mentioned in (B) (*P* < 0.0006 after Bonferroni correction, Fisher’s exact test). Points above the horizontal dashed gray line that have a relative viability <1 are considered statistically significant cytoplasmic–nuclear incompatibilities. The color of a point corresponds to the cytoplasmic genotype being tested. All cytoplasmic genotypes tested show an incompatibility with one or more heterotypic nuclear genotypes. See Figure S4 for separate graphs of all combinations. Above the scatterplot are boxplots depicting the relative viabilities of statistically significant cytoplasmic–nuclear incompatibilities. The color corresponds to the cytoplasmic genotype tested. Incompatibilities involving the NIC59 cytoplasmic genotype have reduced viability compared to those involving the JU1837 and JU1854 cytoplasmic genotypes (*P* < 0.001, Kruskal–Wallis test followed by Dunn’s test). (D) A heatmap depicting the median relative viability for each cytoplasmic–nuclear combination. Each cytoplasmic–nuclear combination shows the proportion of replicates that exhibit significant incompatibilities (for example, three out of three replicates exhibit significant incompatibilities for the NIC59 cytoplasm–JU1854 nuclear combination, while only one out of three replicates exhibit significant incompatibilities for the JU1837 cytoplasm–JU1854 nuclear combination). Each cytoplasmic genotype is consistently incompatible with at least one heterotypic nuclear genotype. The NIC59 cytoplasm has a more distinct response to hybridization than the others tested.

We hybridized additional wild isolates (Figure 3A) to determine whether cytoplasmic–nuclear incompatibilities represent a common reproductive barrier within *C. nouraguensis*, or whether they are an unusual phenotype only observed in hybridizations between NIC59 and JU1825. Specifically, we tested the compatibility of four cytoplasmic genotypes with seven nuclear genotypes. To test for an incompatibility between one strain’s cytoplasm and another strain’s nuclear genome, we again compared the viabilities of backcrosses that differ only in the F1 hybrid female’s cytoplasmic genotype (Figure 3B). Specifically, we compared the viability of the backcross that combines heterotypic cytoplasmic and nuclear genotypes to the viability of the backcross that combines homotypic cytoplasmic and nuclear genotypes. We calculated the relative viability of the two crosses (heterotypic combination/homotypic combination), and tested for statistically significant differences (see *Materials and Methods*). Using the same logic as for our JU1825 × NIC59 crosses, we reasoned that lower viability of the heterotypic cytoplasmic–nuclear combination in comparison to the homotypic cytoplasmic–nuclear combination indicates a cytoplasmic–nuclear incompatibility. Three or four biological replicates were performed for each cytoplasmic–nuclear combination.

Of the 74 cytoplasmic–nuclear tests performed, 50 (67%) exhibited significant incompatibilities (Figure 3C). Additionally, each cytoplasmic genotype was consistently incompatible with at least one heterotypic nuclear genotype (that is, all replicates for a particular cytoplasmic–nuclear combination indicate a significant incompatibility). However, there are a number of cytoplasmic–nuclear combinations whose replicates are inconsistent with one another (that is, some replicates indicate a significant incompatibility while others do not) (Figure 3D and Figure S4). This may indicate that the genetic loci required for hybrid inviability are not fixed between the strains, but rather are polymorphisms segregating within each strain (Cutter 2012; Kozlowska *et al.* 2012; Corbett-Detig *et al.* 2013), consistent with the fact that none of these strains has been formally inbred. Regardless, given their common occurrence in hybridizations between strains of *C. nouraguensis*, we hypothesize that cytoplasmic–nuclear incompatibilities are a significant reproductive barrier within the species.

We generated a heat map to help visualize the median relative viability for each cytoplasmic–nuclear combination (Figure 3D). Strikingly, the NIC59 cytoplasmic genotype exhibits a distinct response to hybridization, being strongly incompatible (that is, having a low median relative viability) with all of the nuclear genotypes tested. By comparison, the other cytoplasmic genotypes can be relatively compatible with some heterotypic nuclear genotypes or exhibit incompatibilities that are typically weaker than those involving the NIC59 cytoplasmic genotype. Specifically, incompatibilities involving the JU1837 or JU1854 cytoplasmic genotypes have significantly higher relative viability (median = 0.72 and 0.71, respectively) in comparison to incompatibilities with the NIC59 cytoplasmic genotype (median = 0.45) (Figure 3C). Incompatibilities involving the JU1825 cytoplasm exhibit an intermediate level of relative viability (median = 0.64) that is statistically indistinguishable from the other cytoplasmic genotypes (*P* = 0.057 in comparison to NIC59; *P* = 1.0 in comparison to both JU1837 and JU1854). Although there is a correlation between the severity of cytoplasmic–nuclear incompatibility and geographic location of the strains hybridized (Figure 3A), too few strains were tested to conclude that the incompatibility studied here has already led to reproductive isolation of these allopatric populations. However, it is clear that the NIC59 cytoplasmic genotype is distinct in terms of the nuclear genotypes it is incompatible with and how severe those incompatibilities are.

### A single BDM incompatibility between a NIC59 cytoplasmic locus and a JU1825 nuclear locus causes embryonic lethality

**Figure 4:**
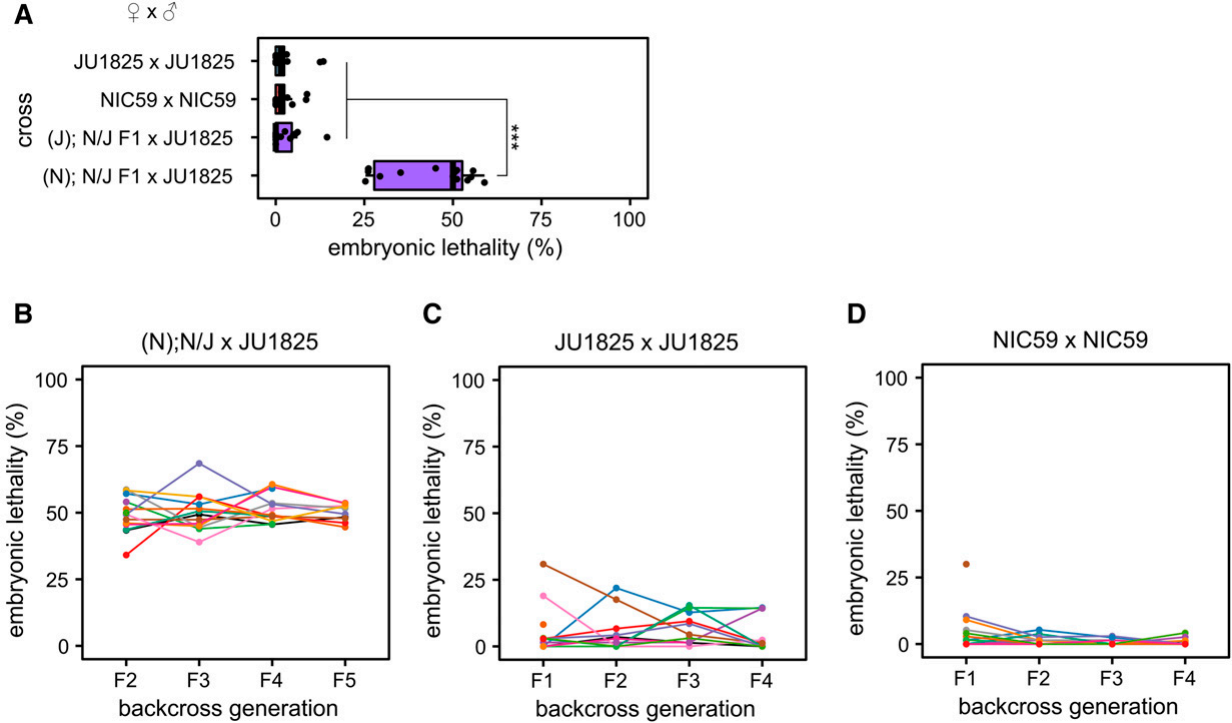
A single BDM incompatibility between a NIC59 cytoplasmic locus and a JU1825 nuclear locus causes embryonic lethality. (A) Approximately 50% of the F2 progeny from (N); N/J F1 female × JU1825 male crosses arrest during embryogenesis, significantly higher than that seen in intrastrain crosses (*P* < 0.001). In contrast, (J); N/J F1 female × JU1825 male and parental strain crosses exhibit similar low levels of embryonic lethality (*P* > 0.05). *N* = 14 or 15 plates per cross. (B) Initially, 15 (N); N/J F1 females were independently backcrossed to single JU1825 males. For each independent lineage, a single surviving F2 female was again backcrossed to a JU1825 male. This backcrossing scheme was repeated until the F5 generation. Each colored line represents a single backcross lineage. All backcross lineages exhibit ∼50% embryonic lethality throughout the backcross generations, consistent with the hypothesis that an incompatibility between a NIC59 cytoplasmic locus and a single JU1825 nuclear locus causes embryonic lethality. Number of independent backcross lineages assayed per generation: F2 = 15, F3 = 13, F4 = 13, F5 = 10. (C) The JU1825 parental strain was “backcrossed” as a negative control. Number of independent backcross lineages assayed per generation: F1 = 15, F2 = 11, F3 = 11, F4 = 10. (D) The NIC59 parental strain was “backcrossed” as a negative control. Number of independent backcross lineages assayed per generation: F1 = 14, F2 = 12, F3 = 12, F4 = 12. All *P*-values were calculated by a Kruskal–Wallis test followed by Dunn’s test.

As previously discussed, the backcross that combines the NIC59 cytoplasmic genotype with JU1825 nuclear genotype (that is, (N); N/J F1 female × JU1825 male, Figure 2B) results in only ∼36% of F2 offspring maturing to the L4 or young adult stage. A more detailed characterization of F2 inviability shows that ∼50% of F2 offspring fail to complete embryogenesis (Figure 4A). Of the remaining half that complete embryogenesis, ∼33% fail to mature to the L4 or young adult stage (data not shown). In comparison, (J); N/J F1 female × JU1825 male crosses result in low levels of embryonic lethality, similar to parental crosses. These data are consistent with F2 embryonic lethality resulting from a single BDM incompatibility between a NIC59 cytoplasmic locus and a single homozygous JU1825 autosomal locus.

To test the hypothesis of a single BDM incompatibility, we crossed F1 (N); N/J females to JU1825 males, then crossed the viable F2 females to JU1825 males and assayed F3 viability. Under this hypothesis, the surviving F2 females are expected to have inherited NIC59 mtDNA and be heterozygous (that is, JU1825/NIC59) at the JU1825 nuclear incompatibility locus (Figure 6A). Therefore, crossing these F2 females to JU1825 males should also result in ∼50% embryonic lethality in the F3 generation. This pattern should also be true for additional backcross generations (F4, F5, *etc*.). Thus, we generated 15 independent backcross lineages, each consisting of matings between single surviving hybrid females and JU1825 males, and monitored each lineage’s viability for four backcross generations. Indeed, the ∼50% embryonic lethality observed in the F2 generation is also observed in the subsequent backcross generations in all lineages (Figure 4B). These results are consistent with the hypothesis that embryonic lethality is the result of a simple two-locus BDM incompatibility between a purely maternally inherited cytoplasmic NIC59 locus and a single nuclear locus homozygous for JU1825 alleles. We hypothesize that the postembryonic inviability may be a genetically separable phenotype.

### The JU1825 cytoplasm appears to be heteroplasmic

As previously discussed, the backcross that combines the JU1825 cytoplasmic genotype with the NIC59 nuclear genotype (that is, (J); N/J F1 female × NIC59 male crosses) results in ∼50% F2 viability on average (Figure 2B). Thus, the total F2 inviability could be the result of a single BDM incompatibility between a JU1825 cytoplasmic locus and a single autosomal locus homozygous for NIC59 alleles.

**Figure 5:**
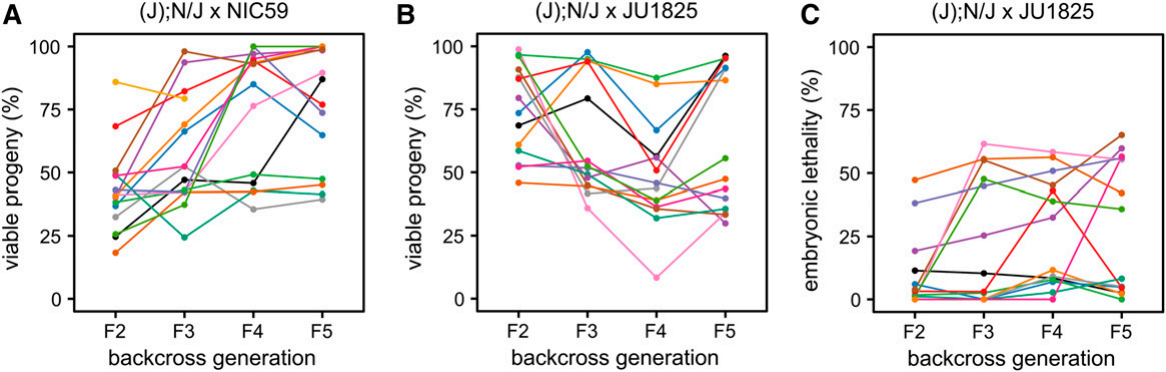
The JU1825 cytoplasm is heteroplasmic for JU1825-like and NIC59-like alleles. (A) The viability of independent (J); N/J female × NIC59 male backcross lineages was followed until the F5 generation. Surprisingly, in some lineages, multiple generations of backcrossing resulted in increased viability (similar to that seen in intrastrain crosses). Number of independent backcross lineages assayed per generation: F2 = 15, F3 = 15, F4 = 14, F5 = 14. (B) The viability of independent (J); N/J female × JU1825 male backcross lineages was also followed until the F5 generation. Interestingly, multiple generations of backcrossing resulted in some lineages with significantly reduced viability, similar to that seen in (N); N/J F1 female × JU1825 male crosses. Number of independent backcross lineages assayed per generation, F2–F5 = 14. (C) Embryonic lethality of the same (J); N/J female × JU1825 male backcross lineages from (B) (with same color-coding). Upon additional generations of backcrossing, some (J); N/J female × JU1825 male lineages exhibit ∼50% embryonic lethality, similar to (N); N/J F1 female × JU1825 male crosses. These results are consistent with the hypothesis that the JU1825 cytoplasm is heteroplasmic and contains JU1825-like and NIC59-like alleles.

To test this hypothesis, we generated 14–15 independent backcross lineages, each consisting of matings between single surviving (J); N/J hybrid females and NIC59 males, and monitored each lineage’s viability for four backcross generations. To our surprise, though some lineages continued to exhibit low levels of viability similar to the F2 generation average (∼50%), others began to exhibit and maintain significantly increased viability for multiple backcross generations (Figure 5A). For example, in this particular experiment we found that in the F2 generation a majority of lineages (13/15) had a total viability ranging from 18 to 50%, while only two exhibited higher viability (68 and 85%). However, by the F5 backcross generation, we found that of the 14 remaining lineages only four exhibited 50% viability or less. Strikingly, by the F5 generation, 5/14 backcross lineages exhibited nearly 100% viability.

The rescue of hybrid inviability for some lineages via several generations of backcrossing is peculiar. One hypothesis to explain this phenomenon is that the JU1825 cytoplasmic or NIC59 nuclear incompatibility loci are not fixed within their respective strains, but rather are segregating polymorphisms (Cutter 2012; Kozlowska *et al.* 2012; Corbett-Detig *et al.* 2013). As a specific example, the JU1825 cytoplasmic incompatibility locus could be heteroplasmic for alleles that are either incompatible or compatible with the NIC59 nuclear genome. The mitochondrial genome is present at a high copy number within a single cell, and it is thought that individual mtDNAs are randomly replicated and segregated to daughter cells during cell division. Studies on the inheritance of various mtDNA heteroplasmies show that their frequency among siblings from the same mother can be highly variable due to the random sampling of mtDNAs and genetic bottlenecks during female germline development (Wallace and Chalkia 2013; Gitschlag *et al.* 2016). Therefore, it is possible that a NIC59-compatible cytoplasmic allele has increased in frequency in some backcross lineages and rescued inviability.

To gain a better understanding of the genetic composition of the JU1825 cytoplasm, we also monitored the viability of (J); N/J female × JU1825 male lineages over four backcross generations. Because this cross combines homotypic JU1825 cytoplasmic and JU1825 nuclear genotypes, we originally predicted that the relatively high rates of F2 viability would persist or possibly increase with additional backcross generations. However, we instead observed that some backcross lineages showed a striking decrease in viability after the F2 generation (Figure 5B). For example, in this particular experiment, lineages in the F2 generation exhibited a uniform distribution of viability, with an average of 74%. By the F5 generation we find two distinct populations of lineages, those with a high viability ranging from 85 to 96% (6/14 lineages) and those with low viability ranging from 29 to 55% (8/14 lineages) (Figure 5B). The latter population has an average viability of 39%, which is similar to that observed in (N); N/J F1 female × JU1825 male crosses (∼36%, Figure 2B), indicating that although these lineages inherited their cytoplasm from JU1825 mothers, they now seem to exhibit low levels of viability similar to those observed in the NIC59 cytoplasmic–JU1825 nuclear incompatibility. One hypothesis to explain these data is that the JU1825 cytoplasm harbors a NIC59-like allele which at a certain threshold frequency can mimic the NIC59 cytoplasmic–JU1825 nuclear incompatibility in certain (J); N/J F1 female × JU1825 male backcross lineages.

**Figure 6:**
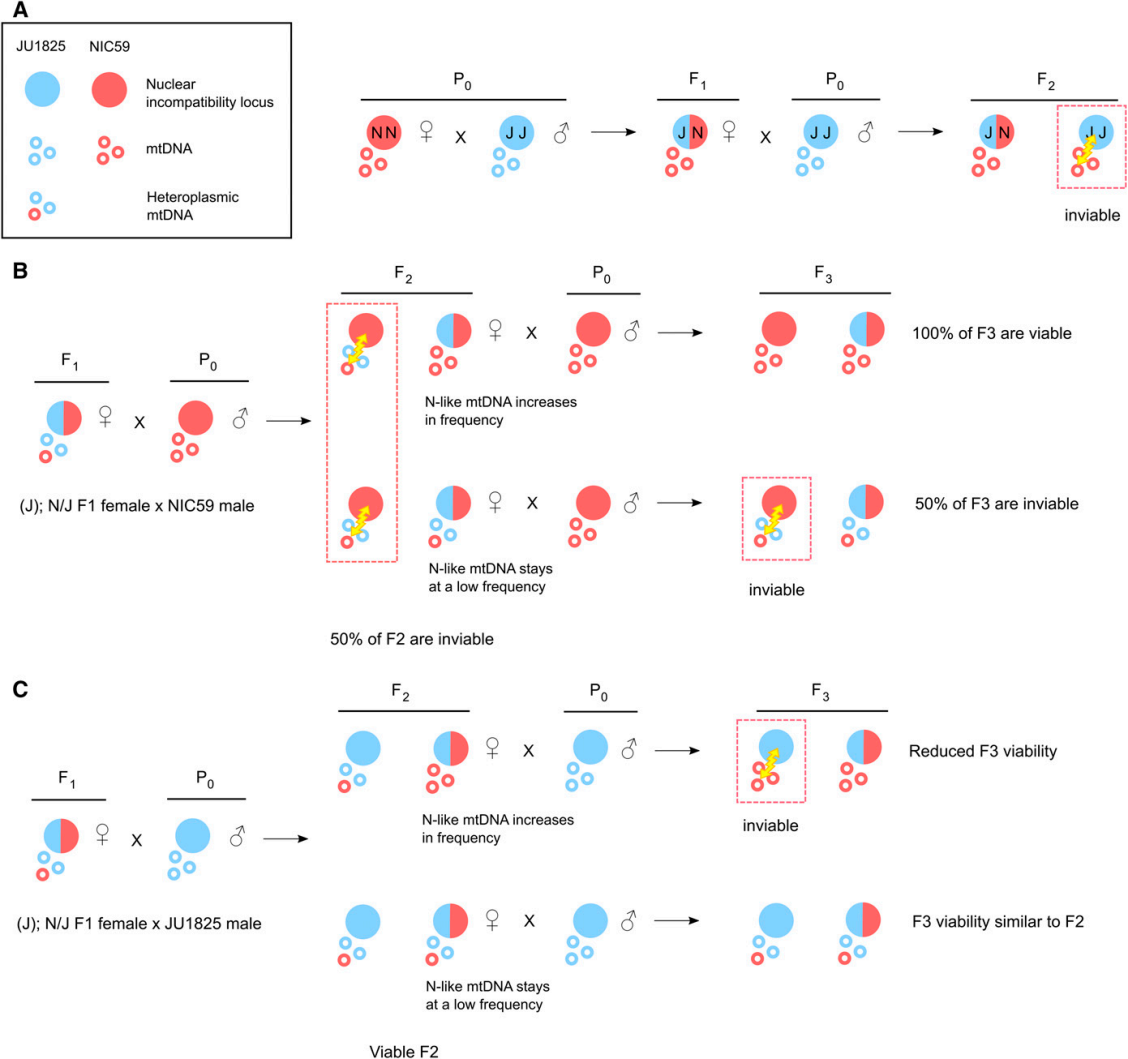
Mitochondrial–nuclear incompatibility model. (A) We hypothesize that F2 hybrid breakdown is the result of a Bateson–Dobzhansky–Muller incompatibility between the NIC59 mitochondrial genome and a nuclear locus homozygous for the JU1825 allele, and vice versa. As a specific example, when NIC59 females are crossed to JU1825 males, the resulting F1 hybrid females are expected to be heterozygous at all autosomal loci but inherit only NIC59 mtDNA. When F1 females are backcrossed to JU1825 males, F2 inviability results from an incompatibility between NIC59 mtDNA and an autosomal locus homozygous for the JU1825 nuclear allele. (B) We hypothesize that the JU1825 cytoplasm is heteroplasmic in F1 females and contains at least one NIC59-like allele. Backcrossing hybrid females with a JU1825 cytoplasm (that is, (J); N/J females) to NIC59 males for multiple generations can allow the NIC59-like cytoplasmic allele to increase in frequency and dilute out the effects of the incompatible JU1825 mtDNA (for example, top right F2 female). This eventually may allow the NIC59 nuclear locus to become homozygous and restore the viability of a lineage. On the other hand, the NIC59-like mtDNA can stay at a low frequency in viable F2 females (for example, bottom right F2 female). Backcrossing these F2 females to NIC59 males results in levels of inviability similar to the F2 generation. (C) By a similar line of reasoning, backcrossing hybrid females with a JU1825 cytoplasm to JU1825 males for multiple generations can allow the NIC59-like mtDNA to increase in frequency, where it can mimic the same genetic incompatibility seen in (N); N/J F1 female × JU1825 male crosses (A).

In support of this hypothesis, the rate of embryonic lethality for some (J); N/J female × JU1825 male backcross lineages also increases to levels observed in the NIC59 cytoplasmic–JU1825 nuclear incompatibility (that is, 50%) and can be stably inherited for several backcross generations (Figure 5C). Specifically, most lineages (12/14) in the F2 generation exhibited only 0–19% embryonic lethality, whereas two lineages exhibited higher rates (38 and 47%). However, by the F5 backcross generation, only about half of the lineages (6/14) exhibited 0–8% embryonic lethality, whereas 8/14 lineages exhibited 35–65% embryonic lethality. Taken together, the results from the two backcross experiments are consistent with the hypothesis that the JU1825 cytoplasm is heteroplasmic and harbors both JU1825-like and NIC59-like incompatibility loci (Figure 6, B and C).

## Discussion

We discovered a lethal cytoplasmic–nuclear incompatibility between two wild isolates of *C. nouraguensis*, JU1825 and NIC59, and find that such incompatibilities may be widespread between other wild isolates within the species. We propose that the mitochondrial genome is the most likely candidate for harboring the cytoplasmic incompatibility factor(s) and further propose that the JU1825 cytoplasm is heteroplasmic and harbors both JU1825-like and NIC59-like incompatibility loci. We show that maternally inherited endosymbiotic bacteria are probably not the cause of hybrid inviability. It remains possible that incompatibility is caused by other cytoplasmically inherited factors (such as maternally inherited small RNAs), or by maternal inheritance of epigenetic marks across several generations.

In eukaryotes, the mitochondrial genome typically contains a very small fraction of the gene content of a cell, yet it seems to be involved in a disproportionate number of genetic incompatibilities across a diverse range of taxa (Rand *et al.* 2004; Burton and Barreto 2012). However, there are relatively few cases in which incompatibility loci have been definitively mapped to the mitochondrial genome, and therefore a larger sample is required to better understand what drives the evolution of mitochondrial–nuclear incompatibility. Additionally, all of the molecularly identified cases of mitochondrial–nuclear incompatibility have been found between species rather than within species (Lee *et al.* 2008; Chou *et al.* 2010; Luo *et al.* 2013; Meiklejohn *et al.* 2013; Ma *et al.* 2016). Some of these interspecies hybridizations harbor additional genetic incompatibilities or chromosomal rearrangements that cause inviability and sterility (Hunter *et al.* 1996; Fischer *et al.* 2000; Brideau *et al.* 2006; Ferree and Barbash 2009; Mihola *et al.* 2009; Davies *et al.* 2016), making it difficult to discern whether mitochondrial–nuclear incompatibility was instrumental in initiating speciation or evolved after strong reproductive isolation occurred. The incompatibility we describe here provides an excellent opportunity to study the evolutionary genetics and cell biology of incipient speciation as well as mitochondrial–nuclear incompatibility. The ease of breeding, large brood sizes, and short generation time of *C. nouraguensis* should facilitate the mapping and identification of the genes that contribute to hybrid inviability.

### Cytoplasmic–nuclear incompatibility: both sexes are equally inviable

J. B. S. Haldane noted that the heterogametic sex more often suffers from inviability or sterility in interspecies hybridizations than the homogametic sex (Delph and Demuth 2016). This rule holds for the handful of recently studied interspecies hybridizations in *Caenorhabditis* (Baird 2002; Woodruff *et al.* 2010; Kiontke *et al.* 2011; Dey *et al.* 2012, 2014; Kozlowska *et al.* 2012; Ragavapuram *et al.* 2016). However, it is not known whether Haldane’s rule also generally applies to intraspecies hybridizations. Interestingly, some intraspecies incompatibilities in *Caenorhabditis* affect both sexes equally (Seidel *et al.* 2008, 2011; Huang *et al.* 2014).

The lethal cytoplasmic–nuclear incompatibility we identified between the NIC59 and JU1825 wild isolates of *C. nouraguensis* also affects females and males equally, suggesting that the two sexes share the same disrupted developmental pathway(s). However, we have not carefully studied other aspects of sex-specific fitness, such as female and male F2 hybrid fertility. Because the mitochondrial genome is inherited only through females, theory predicts that evolution will lead to the accumulation of mtDNA variants that are neutral or increase female fitness, but that are neutral or possibly deleterious to male fitness (Gemmell *et al.* 2004; Patel *et al.* 2016). Thus, male-specific functions may be more adversely affected during the hybridization of heterotypic mitochondrial and nuclear genomes. This is indeed the case for some known mitochondrial–nuclear incompatibilities. For example, when swapping the mitochondrial genomes between mouse subspecies via pronuclear transfer, one mitochondrial–nuclear combination resulted in reduced male fertility whereas females had relatively normal fertility (Ma *et al.* 2016). Therefore, further studies of *C. nouraguensis* hybrid male fertility will be required to more fully address whether this system follows Haldane’s rule, as well as to determine whether there are male-specific mitochondrial–nuclear incompatibilities.

### Symmetric cytoplasmic–nuclear incompatibilities in C. nouraguensis

Reciprocal interspecific crosses often show differences in the viability or fertility of hybrids. This asymmetry in hybrid fitness (termed “Darwin’s corollary” to Haldane’s rule) has been theorized to be the result of uniparentally inherited factors from one species (such as maternal RNAs, sex chromosomes, or cytoplasmically inherited genomes), being incompatible with heterospecific loci of the other, but not vice versa (Turelli and Moyle 2007). Darwin’s corollary is also seen in several hybridizations in the *Caenorhabditis* genus, probably due to X-linked incompatibilities (Woodruff *et al.* 2010; Dey *et al.* 2012, 2014; Kozlowska *et al.* 2012; Ragavapuram *et al.* 2016).

Consistent with Darwin’s corollary to Haldane’s rule, most molecularly characterized BDM incompatibilities are asymmetric, in that only one of two divergent alleles at a locus is incompatible with heterospecific alleles at other loci (Brideau *et al.* 2006; Ferree and Barbash 2009). This is also true of the asymmetric mitochondrial–nuclear incompatibilities seen in *Saccharomyces* species hybridizations (Lee *et al.* 2008; Chou *et al.* 2010). For example, an intron of the *COX1* gene in the *Saccharomyces bayanus* mitochondrial genome fails to be correctly spliced by the nuclearly encoded *S. cerevisiae MRS1* gene, resulting in hybrid inviability. However, a similar incompatibility does not occur between *S. cerevisiae COX1* and *S. bayanus MRS1*. In our study, despite differences in severity, cytoplasmic–nuclear incompatibilities involving NIC59 appear to be symmetric (Figure 2B and Figure 3D). However, with our current data, we cannot determine whether the same or different genes cause hybrid inviability in the reciprocal crosses. Multiple distinct cytoplasmic–nuclear incompatibilities between these strains might be an indication of rapid divergent cytoplasmic–nuclear coevolution within the species.

### JU1825 heteroplasmy

We hypothesize that the JU1825 cytoplasm is heteroplasmic and contains mitochondrial genomes that are both compatible (JU1825-like) and incompatible (NIC59-like) with the JU1825 nuclear incompatibility locus. If the JU1825 cytoplasm is naturally heteroplasmic, we predict the NIC59-like mtDNAs are kept at a low frequency within JU1825 by selection. This selection would be relaxed in (J); N/J F1 hybrids and the frequency of NIC59-like mtDNA could increase beyond a certain threshold, reducing incompatibility in backcrosses to NIC59 males and increasing incompatibility in backcrosses to JU1825 males. However, another possibility is that NIC59-like mtDNA is introduced into F1 females by incomplete degradation and inheritance of paternal NIC59 mtDNA. Interestingly, evidence suggests that paternal mtDNA can be inherited when hybridizing different wild isolates of *Caenorhabditis briggsae* (Hicks *et al.* 2012; Chang *et al.* 2015; Ross *et al.* 2016).

The hypothesized heteroplasmy may explain the greater variance of F2 viability in crosses with (J); N/J F1 females in comparison to those with presumably homoplasmic (N); N/J F1 females. Stochastic segregation and genetic bottlenecking events from JU1825 mothers (or variable paternal leakage from NIC59 fathers) may result in F1 females with a wide range of frequencies of the NIC59-like cytoplasmic allele, and therefore a wide range of F2 viability when backcrossed to either NIC59 or JU1825 males. Such stochastic inheritance could explain why the degree of F2 viability of (J); N/J F1 female × JU1825 male backcrosses can also vary significantly from experiment to experiment (Figure S1).

### Caenorhabditis nematodes as models to study speciation

The nematodes of the *Caenorhabditis* genus are currently emerging as a model system for the genetic study of hybrid incompatibility. Previous studies were restricted by the limited number of known species and wild isolates. However, the recent discovery that *Caenorhabditis* nematodes are found primarily in rotting fruits has led to a continuously expanding number of wild isolates of known and new species, greatly increasing the number of crosses in which intra- and interspecies incompatibilities can be studied (Kiontke *et al.* 2011).

Studies of genetic incompatibilities between well-defined species often reveal that many genetic variants contribute to hybrid dysfunction, making it difficult to discern which initially decreased gene flow and which evolved after strong reproductive barriers had evolved. On the other hand, incomplete reproductive barriers between different populations of the same species may or may not be indicative of incipient speciation. Therefore, to understand the accumulation of postzygotic isolating barriers, one would ideally monitor the same two divergent lineages throughout the entire speciation process (Seehausen *et al.* 2014). This is impractical for most multicellular organisms. An alternative method is to compare and contrast hybridizations with differing degrees of postzygotic isolation across the species continuum, ranging from weak postzygotic isolation within species to strong postzygotic isolation between distinct species.

The *Caenorhabditis* genus has the potential to span such a continuum. Interestingly, both *C. briggsae* and *C. nouraguensis* appear to have intraspecies cytoplasmic–nuclear incompatibilities (Ross *et al.* 2011; Chang *et al.* 2015). Although the exact genetic components of these incompatibilities have not been identified, these two cases add to an already large literature of cytoplasmic–nuclear incompatibilities, implying a role for divergent cytoplasmic–nuclear coevolution in driving speciation. Near the other end of the species continuum, hybridizations between the well-defined sister-species *C. briggsae* and *C. nigoni* produce a low degree of F1 embryonic lethality and either hybrid male sterility or inviability, depending on the cross direction (Woodruff *et al.* 2010; Kozlowska *et al.* 2012; Ragavapuram *et al.* 2016). In contrast to the relatively simple intraspecies genetic incompatibilities in *C. nouraguensis*, *C. briggsae*, and *C. elegans* (Seidel *et al.* 2008, 2011; Ross *et al.* 2011; Baird and Stonesifer 2012), a recent genome-wide introgression study revealed the presence of many distinct *C. briggsae* loci that are sufficient to cause hybrid dysfunction in an otherwise *C. nigoni* background (Bi *et al.* 2015). Future identification and comparison of genes required for hybrid inviability or sterility across the *Caenorhabditis* speciation continuum may give insight into the evolutionary forces that promote speciation.

## Supplementary Material

Supplemental material is available online at www.g3journal.org/lookup/suppl/doi:10.1534/g3.116.037101/-/DC1.
