# Supplementary material for: Cytoplasmic–Nuclear Incompatibility Between Wild Isolates of Caenorhabditis nouraguensis
Source: G3 (Bethesda). 2017 Jan 5;7(3):823–34. doi: 10.1534/g3.116.037101 (PMC5345712; doi:10.1534/g3.116.037101)
Supplement: Supplementary file 1 [file 823FigureS1.pdf]

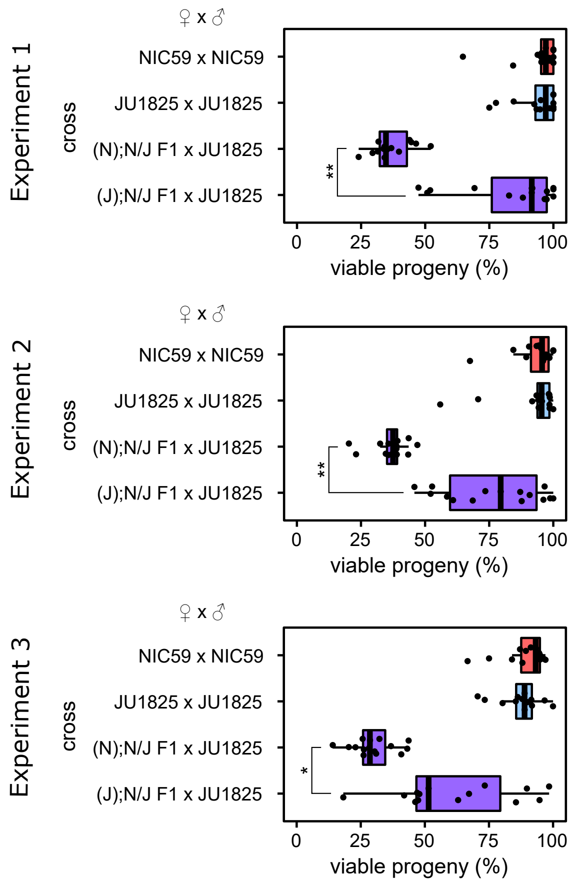

Figure S1. Variability of (J); N/J F1 female x JU1825 male crosses across experiments.
Three biological replicates of the same type of backcross experiment. (J); N/J F1 female x JU1825 male crosses can either exhibit similar or significantly decreased rates of viability in comparison to intra-strain crosses (Experiment 1, non-significant, P>0.05; Experiment 2, non-significant, P>0.05; Experiment 3, P>0.05, non-significant in comparison to JU1825 x JU1825 crosses, P<0.05 significant in comparison to NIC59 x NIC59 crosses). However, (J); N/J F1 female x JU1825 male crosses consistently exhibit significantly increased rates of viability in comparison to (N); N/J female x JU1825 male crosses (Experiment 1, **, P<0.01; Experiment 2, **, P<0.01; Experiment 3, *, P<0.05). Experiments 1 and 2 are data from Figures 2 and 5, respectively. All p-values were calculated by a Kruskal-Wallis test followed by Dunn's test.
