# Supplementary material for: Cytoplasmic–Nuclear Incompatibility Between Wild Isolates of Caenorhabditis nouraguensis
Source: G3 (Bethesda). 2017 Jan 5;7(3):823–34. doi: 10.1534/g3.116.037101 (PMC5345712; doi:10.1534/g3.116.037101)
Supplement: Supplementary file 2 [file 823FigureS2.pdf]

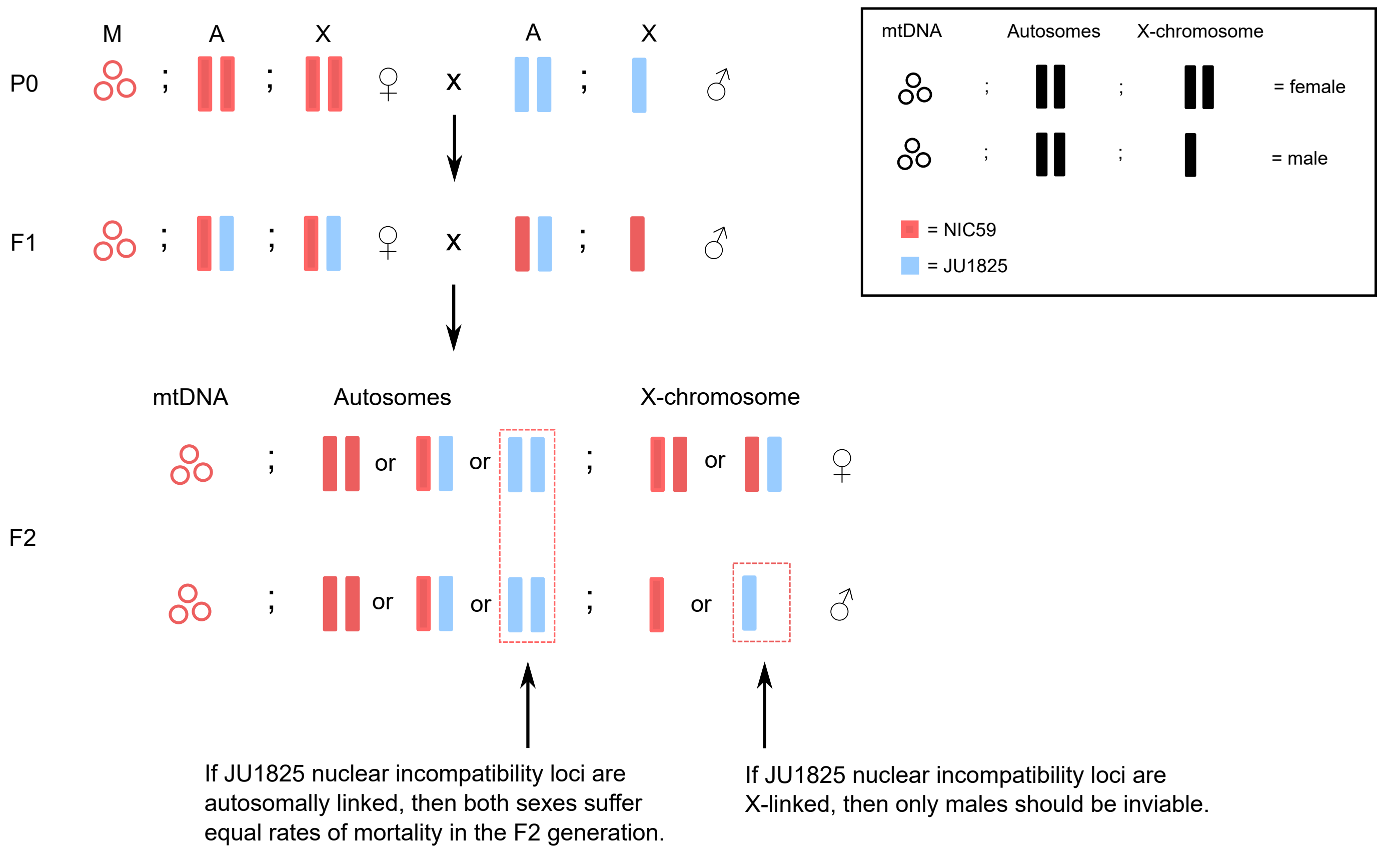

Figure S2. Nuclear incompatibility loci are linked to autosomes, not sex chromosomes.
F1 intercrosses allow us to infer that the nuclear incompatibility loci are autosomal, not X-linked. From the (N); N/J F1 x JU1825 male backcross experiment (Figure 2), we concluded that F2 inviability was the result of a genetic incompatibility between the NIC59 mitochondrial genome and nuclear loci homozygous for JU1825 alleles. It is reasonable to assume that the same genetic incompatibility contributes to F2 inviability in (N); N/J F1 female x (N); N/J F1 male crosses. If the nuclear incompatibility locus were X-linked, F2 male progeny of F1 intercrosses would have a 50% chance of being hemizygous for the JU1825 nuclear incompatibility locus whereas F2 females would only be heterozygous or homozygous for NIC59 alleles. Therefore, if the locus were X-linked, half of the F2 males would be inviable while females would be unaffected. If the nuclear incompatibility locus were autosomally linked, then both sexes would have an equal chance of being homozygous for the JU1825 nuclear incompatibility locus and thus, both sexes would be expected to suffer equal rates of inviability. We do not observe a significant decrease in the proportion of viable F2 males (Figure 1), so we conclude that the JU1825 nuclear incompatibility locus or loci are linked to autosomes. The same line of reasoning can be used to show that the NIC59 incompatibility locus or loci are also autosomally linked.
