# Supplementary material for: Cytoplasmic–Nuclear Incompatibility Between Wild Isolates of Caenorhabditis nouraguensis
Source: G3 (Bethesda). 2017 Jan 5;7(3):823–34. doi: 10.1534/g3.116.037101 (PMC5345712; doi:10.1534/g3.116.037101)
Supplement: Supplementary file 3 [file 823FigureS3.pdf]

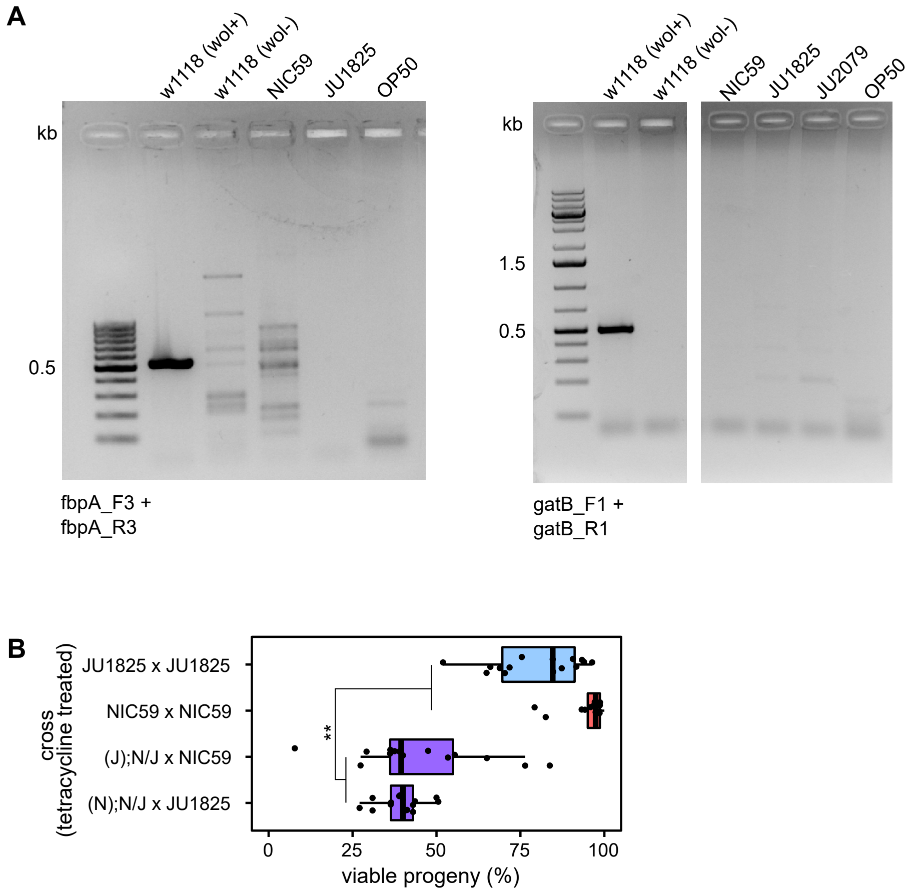

Figure S3. Endosymbiotic bacteria do not cause cytoplasmic-nuclear incompatibility.
(A) PCR on both JU1825 and NIC59 crude lysates (10 adult worms per lysate, 5 females and 5 males) with degenerate primers against *Wolbachia fbpA* or *gatB* loci fails to amplify the expected products. w^{1118} (wol+) and w^{1118} (wol-) *D. melanogaster* flies serve as positive and negative controls, respectively. PCR on crude lysates of OP50 (bacterial food source of NIC59 and JU1825) also fails to amplify the expected products. PCR on JU2079, an inbred strain derived from JU1825, also fails to amplify the expected *gatB* product. **(B)** After tetracycline treatment, both (J); N/J F1 female x NIC59 male and (N); N/J female x JU1825 male crosses still exhibit significantly decreased levels of viability in comparison to tetracycline-treated intra-strain crosses ($P<0.01$). Additionally, there are no statistical differences in viability between NIC59 x NIC59 and JU1825 x JU1825 tetracycline treated intra-strain crosses ($P>0.05$). N=14 or 15 for each cross. All p-values were calculated by a Kruskal-Wallis test followed by Dunn's test.
