# Supplementary material for: Cytoplasmic–Nuclear Incompatibility Between Wild Isolates of Caenorhabditis nouraguensis
Source: G3 (Bethesda). 2017 Jan 5;7(3):823–34. doi: 10.1534/g3.116.037101 (PMC5345712; doi:10.1534/g3.116.037101)
Supplement: Supplementary file 4 [file 823FigureS4.pdf]

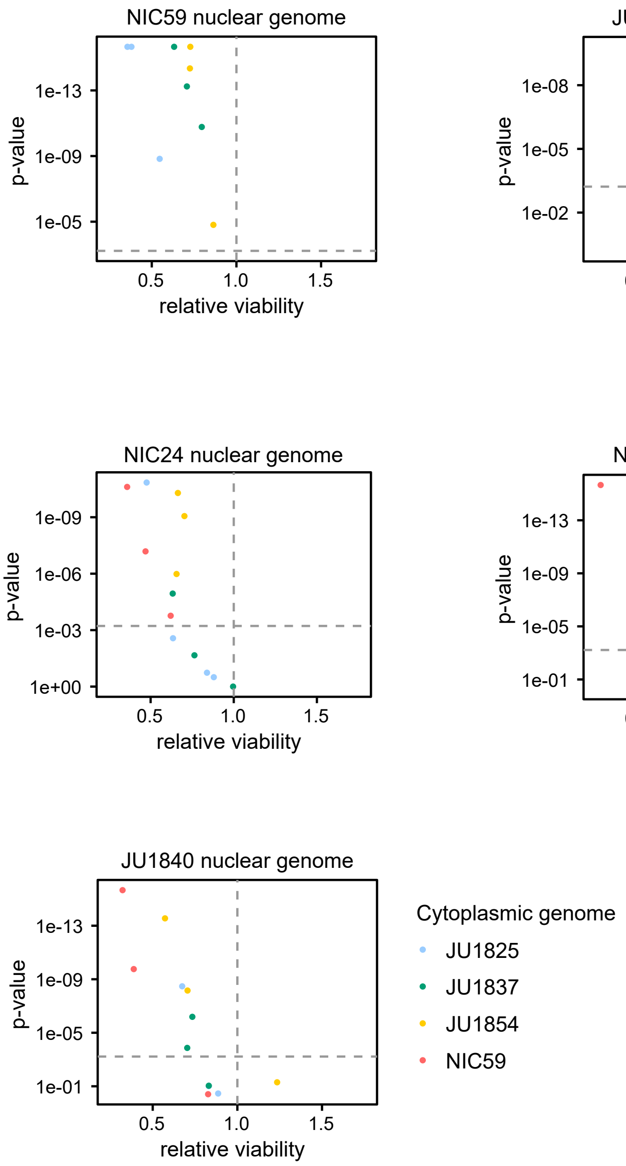


**Figure S4. Cytoplasmic-nuclear tests of different *C. nouraguensis* strains.**
Each graph depicts all the cytoplasmic-nuclear tests performed between four cytoplasmic genotypes and a single nuclear genotype. This is the same data that is grouped into a single graph in Figure 3C. Each cytoplasmic-nuclear combination has three biological replicates (except for JU1825 cytoplasm - NIC24 nuclear and JU1825 cytoplasm - NIC54 nuclear combinations, which have four replicates). Although there appear to be many cases of significant cytoplasmic-nuclear incompatibility (relative viability<1 and P<0.0006 after Bonferroni correction), there can be discrepancies between replicates (for example, one replicate of the JU1825 cytoplasm - NIC24 nuclear combination indicates a significant incompatibility, while the other three do not).
